# The impact of small-sided games on the athletic performance of basketball players: a systematic review and meta-analysis of randomized controlled trials

**DOI:** 10.3389/fpsyg.2026.1799413

**Published:** 2026-06-26

**Authors:** Lang Wang, Haoming Yan, Guangjie Xin

**Affiliations:** 1 School of Sports Training, Chengdu Sport University, Chengdu, China; 2 School of Physical Education, Chengdu Sport University, Chengdu, China; 3 College of Physical Education and Health Sciences, Zhejiang Normal University, Jinhua, China

**Keywords:** athletic performance, basketball, meta-analysis, small-sided games, systematic review

## Abstract

**Objective:**

Small-sided games (SSG), as a game-based training approach, offer unique advantages compared with traditional training methods. However, there is currently no consensus regarding the effects of SSG versus traditional training on athletic performance in basketball players. Therefore, the purpose of this study was to evaluate the effects of small-sided training on athletic performance in basketball players.

**Methods:**

A systematic search was conducted in Web of Science, EBSCO, PubMed, Embase, and the Cochrane Library from database inception to January 3, 2026. Randomized controlled trials investigating the effects of SSG on basketball players’ athletic performance were included. Outcomes related to physical performance (aerobic performance, sprinting, agility, and jumping) and basketball-specific technical performance (shooting, passing, and dribbling) were extracted. Meta-analyses were performed using fixed- or random-effects models based on heterogeneity, and risk of bias was assessed using the Cochrane framework.

**Results:**

Nine randomized controlled trials involving 180 basketball players were included. The meta-analysis showed that, compared with traditional training, SSG significantly improved agility, shooting, passing and dribbling. However, no significant differences were found between SSG and control interventions for aerobic performance, sprinting, or jumping.

**Conclusion:**

Small-sided games are an effective training strategy for enhancing agility and basketball-specific technical performance, but they do not demonstrate clear advantages over traditional training in improving aerobic performance, sprint performance, or jumping ability. SSG may serve as a valuable complement to conventional training methods, particularly for developing technical and tactical abilities, but they should not replace traditional physical conditioning programs.

**Systematic review registration:**

CRD420261285674.

## Introduction

Basketball is a high-intensity, intermittent team sport characterized by intense physical contact and rapid transitions between offense and defense (Castillo et al., 2021). During competition, players must quickly perceive constantly changing on-court information and make real-time tactical decisions, while repeatedly performing complex movements such as sprinting, jumping, rapid changes of direction, and physical contact (de Pedro-Múñez et al., 2025; Rösch et al., 2021). Consequently, athletes’ athletic performance is a decisive factor influencing match outcomes in basketball. With the continuous advancement of global basketball standards, increasingly higher demands have been placed on players’ athletic performance, making the long-term optimization of athletic performance a central focus of basketball training programs (Usgu et al., 2020).

Traditionally, improvements in basketball performance have relied heavily on structured physical conditioning, sport-specific technical drills, and full-court competitive matches. Although these methods can effectively enhance specific performance components, they often require a trade-off between training specificity and load regulation (Palmer et al., 2021). While full-court match play is essential, it may also lead to potential negative effects, such as excessive physiological and psychological loads on players and limited individual ball involvement, thereby reducing opportunities for individual performance expression (Pernigoni et al., 2024; Abdelkrim et al., 2010). To some extent, these factors may constrain the comprehensive development of basketball players (García-Ceberino et al., 2022). Consequently, coaches and practitioners have increasingly explored alternative competition formats that preserve the authenticity of the game while allowing more precise regulation of training and competitive demands. In this context, small-side games (SSG) have emerged as a novel competition-based training approach. By reducing playing area dimensions or the number of participants, implementing flexible rules, and manipulating recovery periods, SSG aims to enhance basketball players’ sport-specific technical skills and physical fitness (Halouani et al., 2014; Clemente, 2016).

In recent years, an increasing number of experimental studies have investigated the effects of SSG on basketball players (Rodríguez-Fernández et al., 2025; Li et al., 2024). However, findings across studies remain inconsistent. While some studies have demonstrated that SSG leads to greater performance improvements than traditional training or regular practice, others have reported comparable or limited effects (Brini et al., 2020; Zeng et al., 2022; Arslan et al., 2022). Differences in athlete characteristics, competitive level, intervention duration, game formats, and outcome assessments may partially account for these discrepancies.

Despite the growing application of SSG in basketball training, the existing evidence regarding their effects on athletic performance remains fragmented and lacks systematic integration. To date, no systematic review and meta-analysis has specifically examined the impact of small-side games on athletic performance in basketball players. Consequently, the overall effect size and consistency of SSG on key athletic performance outcomes in basketball remain unclear. The objective of this research was to conduct a systematic review and meta-analysis of the existing evidence regarding the impact of small-side games on basketball players’ athletic performance. This aims to offer evidence-based recommendations for coaches, strength and conditioning specialists, and researchers who are looking to enhance strategies for the development of basketball performance.

## Methods

### Registration

This systematic review was registered in advance in the PROSPERO database (CRD420261285674, registered on January 14, 2026) and was carried out in full compliance with the PRISMA guidelines (Page et al., 2021).

### Search strategy

A systematic search was conducted across five major databases, including Web of Science, EBSCO, PubMed, Embase, and the Cochrane Library, encompassing all records from the database’s inception to January 3, 2026. The search strategy was developed around the core terms “small-sided games,” “basketball,” and “randomized controlled trial,” and was expanded using related keywords and synonyms. To identify additional publications that met our review’s eligibility criteria, we carefully examined the reference lists of relevant studies. Through this reference screening process, we expanded the search scope and identified potentially relevant articles that may have been missed in the initial search. The detailed search strategy is provided in the Supplementary material.

### Eligibility criteria

The eligibility criteria were structured according to the PICOS framework as follows: Population: basketball players, without restrictions on sex, age, or competitive level; Intervention: small-sided games-based training, conducted at least once a week, with no time limit on the duration; Comparator: training approaches other than small-sided games, including high-intensity interval training (HIIT) or traditional basketball training (TBT); Outcomes: athletic performance outcomes, including aerobic performance, sprinting, agility, jumping, shooting, passing, and dribbling; Study design: randomized controlled trials (Table 1).

**Table 1:** PICOS formats.

Category	Inclusion criteria	Exclusion criteria
Population	Basketball players	Basketball players with severe injuries or serious diseases
Intervention	Small-sided games-based training, conducted at least once a week, with no time limit on the duration	Training approaches other than small-sided games
Comparator	Training approaches other than small-sided games	Studies without a control group
Outcome	Athletic performance outcomes, including aerobic performance, sprinting, agility, jumping, shooting, passing, and dribbling	No athletic performance outcomes
Study design	RCTs	Non-RCTs

The exclusion criteria were: (1) basketball players with severe injuries or serious diseases; (2) studies without a control group; (3) studies that did not report athletic performance-related outcome measures; (4) non-RCTs; and (5) conference abstracts, theses or dissertations, book chapters, letters, or patent publications.

### Study selection and data extraction

The literature screening and data extraction processes were carried out independently by two researchers. Any disputes that arose were resolved through discussions with a third researcher. To guarantee the exclusion of irrelevant papers, the titles and abstracts were reviewed in the first screening phase. Following this, the complete texts of the articles that passed the initial review were evaluated, resulting in the elimination of those that did not meet the predetermined inclusion and exclusion criteria.

Extracted information included study characteristics (authors, publication year), participant characteristics (sample size, age), control intervention characteristics, and outcome measures related to athletic performance. In addition, to better characterize the SSG interventions and facilitate interpretation of between-study heterogeneity, other features were extracted when available, including player configuration, court dimensions, intervention duration, session frequency, and other contextual constraints.

### Study quality assessment

As per the guidelines outlined in the Cochrane Handbook for Systematic Reviews of Interventions, the evaluation of bias risk was conducted independently by two researchers. When disagreements arose, a third researcher was consulted to achieve consensus. The certainty of evidence for each outcome was assessed using the GRADE approach.

### Statistical analysis

Statistical analyses of continuous variables related to athletic performance were performed using Review Manager software. For each study, 95% confidence intervals (CIs) were calculated based on the mean values and standard deviations (SDs) of the relevant outcomes (Deeks et al., 2019). When outcome measures were assessed using different methods or units, effect sizes were expressed as standardized mean differences (SMDs); otherwise, weighted mean differences (WMDs) were calculated (Higgins et al., 2019). Between-study heterogeneity was evaluated using the *I*^2^ statistic. A fixed-effects model was applied when heterogeneity was low (*p* > 0.1 and *I*^2^ < 50%); in all other cases, a random-effects model was used. Given the expected clinical and methodological heterogeneity among SSG interventions (e.g., variations in player configuration, training duration, and contextual constraints), the random-effects model was considered more appropriate when heterogeneity was present. Sensitivity analyses were conducted using a leave-one-out approach (Viechtbauer and Cheung, 2010); if the pooled results changed substantially after removal of a single study, that study was considered to have a significant influence on the overall findings. Publication bias was assessed using funnel plots and Egger’s test when ≥10 studies were available.

For clarity of interpretation, the direction of outcomes was defined according to clinical performance meaning. For time-based measures (e.g., agility and sprinting), lower values represent better performance; therefore, negative SMD values favor SSG. For outcomes expressed as scores, distances, or accuracy percentages (e.g., shooting, passing, jumping), higher values represent better performance.

## Results

### Literature search results

**Figure 1:**
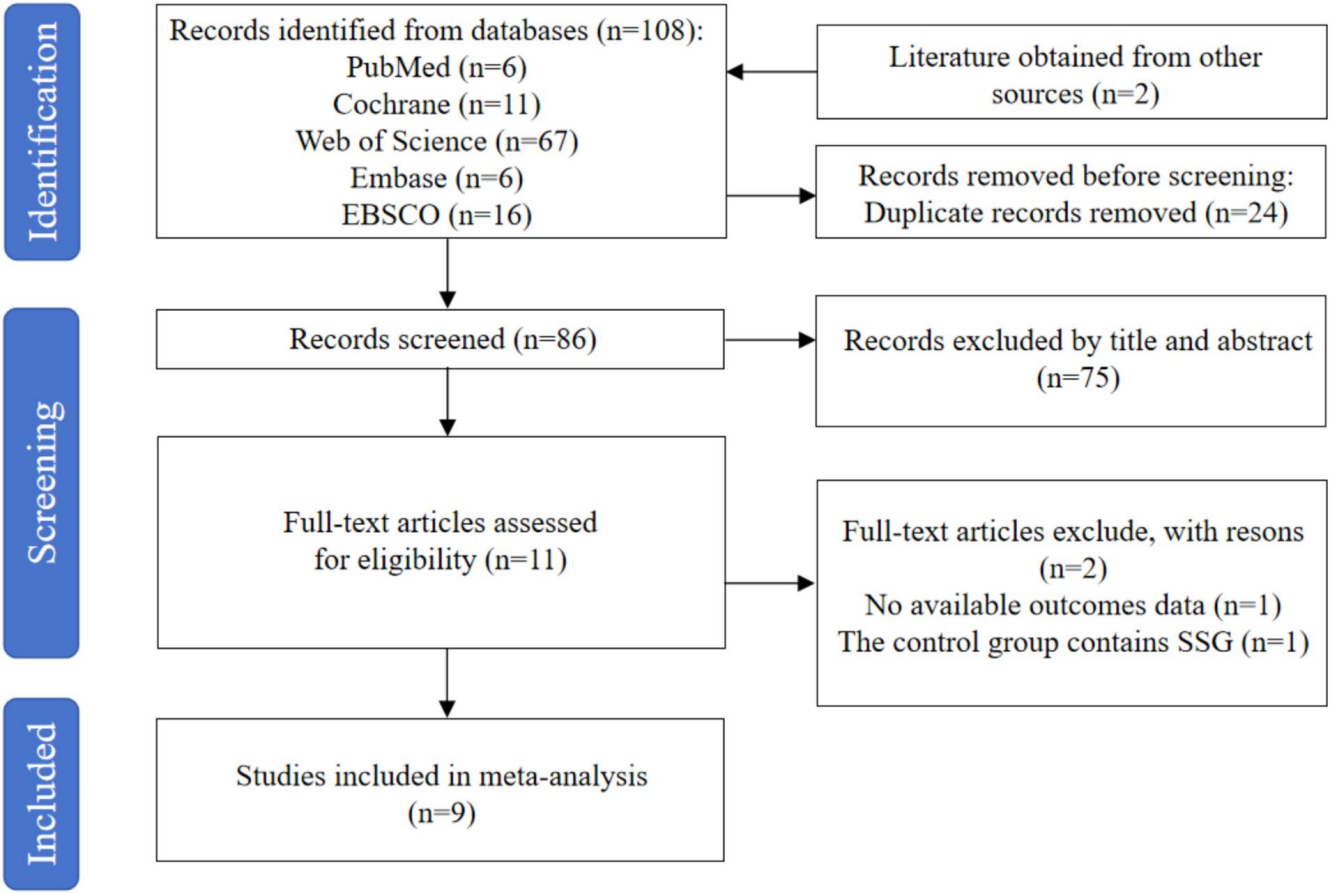
Flow diagram of literature selection process.

The process for selecting studies is illustrated in Figure 1. From five databases, 106 records were identified, with an additional two records sourced from other avenues, leading to a cumulative total of 108 records for review. After the elimination of 24 duplicates, 75 records were rejected based on the screening of titles and abstracts. The full texts of the records that remained were subsequently evaluated, resulting in the exclusion of two studies for failing to satisfy the eligibility requirements. In the end, 9 studies were found to be eligible and included in the final analysis.

### Basic characteristics of the included literature

The nine randomized controlled trials included in this review were published between 2014 and 2024 (Brini et al., 2020; Zeng et al., 2022; Arslan et al., 2022; Delextrat and Martinez, 2014; Delextrat et al., 2018; Maggioni et al., 2019; Gomes et al., 2021; Hassan et al., 2023; Xu et al., 2024). Collectively, these studies involved 180 basketball players aged 10–27 years. In most studies, the duration of the training intervention exceeded 3 weeks. Regarding physical performance outcomes, eight studies reported changes in aerobic performance and agility, seven studies reported sprint speed, and six studies reported jump performance. In terms of basketball-specific technical performance, five studies reported outcomes related to shooting and passing, while four studies reported outcomes related to dribbling performance. The key characteristics of the included studies are presented in Table 2.

**Table 2:** Characteristics of eligible studies.

Included studies	Sample size (N)	Age of participants	Intervention		SSG format	Court Dimensions (m × m)	Control intervention	Outcome measures
			E	C				
Delextrat and Martinez (2014)	E = 9, C = 9	EG: 16.0 ± 0.6 CG: 16.3 ± 0.8	SSG	HIIT	2 V2	28 × 7.5	6 weeks, 2 sets×2reps × 3 min with 45 s rest, 3 times/week	Aerobic performance, sprinting, shooting, passing, agility
Delextrat et al. (2018)	E = 10, C = 10	14.3 ± 0.6	SSG	HIIT	2 V2	28 × 7.5	6 weeks, 2 sets×2reps × 3 min with 45 s rest	Aerobic performance, sprinting
Maggioni et al. (2019)	E = 12, C = 9	19.0 ± 1.0	SSG	TBT	3 V3	Half court	8 weeks, 3 reps×4 min with 60 s rest,3 times/week	Aerobic performance, sprinting, shooting, passing, jumping, agility, dribbling
Brini et al. (2020)	E = 8, C = 8	23.4 ± 2.3	SSG	HIIT	2 V2	28 × 7.5	4 weeks, 2 sets×2 reps × 3 min with 45 s rest, 2 times/week	Jumping, agility
Gomes et al. (2021)	E = 5, C = 5	EG: 23.8 ± 5.0 CG: 26.6 ± 3.3	SSG + TBT	TBT	3 V3	Half court	6 weeks, 7 times/week	Aerobic performance, sprinting, jumping, agility
Arslan et al. (2022)	E = 16, C = 16	EG: 14.4 ± 0.5 CG: 14.6 ± 0.5	SSG	HIIT	2 V1	Full court	6 weeks, 2 sets×2 reps × 3 min with 120 s rest，3 times/week	Aerobic performance, sprinting, shooting, passing, jumping, agility, dribbling
Zeng et al. (2022)	E = 9, C = 10	EG: 20.0 ± 1.3 CG: 19.8 ± 0.8	SSG	HIIT	2 V2	15 × 14	6 weeks, 3 sets × 2 reps × 3 min with 45 s rest，3 times/week	Aerobic performance, sprinting, shooting, passing, jumping, agility, dribbling
Hassan et al. (2023)	E = 12, C = 12	10.9 ± 0.8	SSG	TBT	Not reported	Not reported	10 weeks, 70-min, 4 times/week	Shooting, passing, agility, dribbling
Xu et al. (2024)	E = 10, C = 10	EG: 20.0 ± 1.3 CG: 19.8 ± 0.8	SSG	TBT	3 V3	28 × 7.5	6 weeks, 30-min, 3 times/week	Aerobic performance, sprinting, jumping, agility

E, experimental group; C, control group; HIIT, high-intensity interval training; TBT, traditional basketball training.

### Study quality assessment

**Figure 2:**
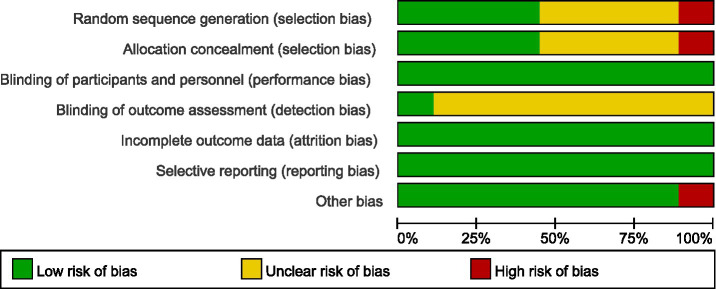
Overall overview graph of bias risk in included studies.

**Figure 3:**
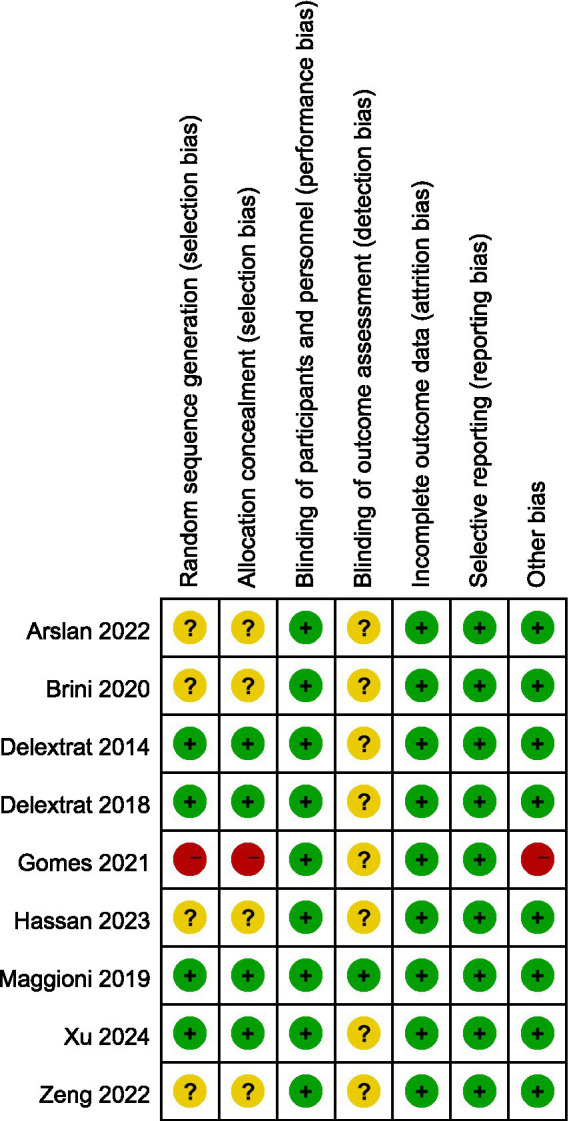
Risk of bias evaluation graph for the included literature.

Figures 2, 3 present the assessment results of bias risk. Overall, the included trials demonstrated relatively satisfactory methodological quality, providing reliable evidence for this review. According to the GRADE assessment, the certainty of evidence ranged from moderate to low across outcomes.

### Meta-analysis

#### Aerobic performance

**Figure 4:**
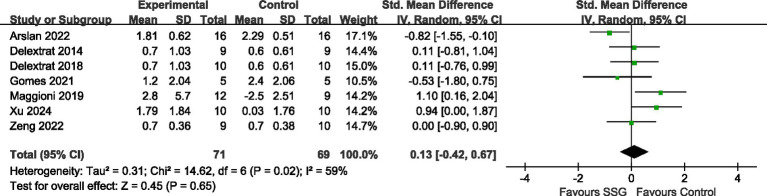
Forest plot of SSG intervention on aerobic performance.

The results of the meta-analysis using a random-effects model showed that, compared with the control group, the SSG group did not demonstrate a statistically significant difference (SMD = 0.13, 95% CI: −0.42 to 0.67, *p* = 0.65) (Figure 4).

#### Sprinting

**Figure 5:**
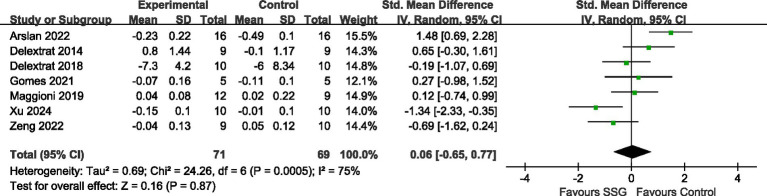
Forest plot of SSG intervention on sprinting.

Regarding sprinting, the results indicated that there was no statistically significant difference between the SSG group and the control group (SMD = 0.06, 95% CI: −0.65 to 0.77, *p* = 0.87) (Figure 5).

#### Agility

**Figure 6:**
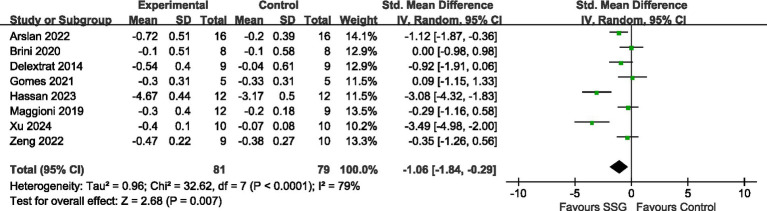
Forest plot of SSG intervention on agility.

Regarding agility, the meta-analysis results showed a statistically significant difference between the SSG group and the control group (SMD = −1.06, 95% CI: −1.84 to −0.29, *p* = 0.007) (Figure 6).

#### Jumping

**Figure 7:**
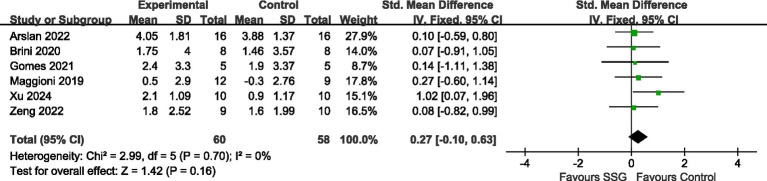
Forest plot of SSG intervention on jumping.

Regarding jump performance, no statistically significant difference was observed between the SSG group and the control group (SMD = 0.27, 95% CI: −0.10 to 0.63, *p* = 0.16) (Figure 7).

#### Shooting

**Figure 8:**
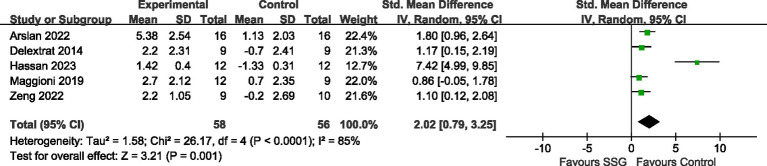
Forest plot of SSG intervention on shooting.

Regarding shooting performance, the meta-analysis based on a random-effects model demonstrated a statistically significant difference between the SSG group and the control group (SMD = 2.02, 95% CI: 0.79 to 3.25, *p* = 0.007) (Figure 8).

#### Passing

**Figure 9:**
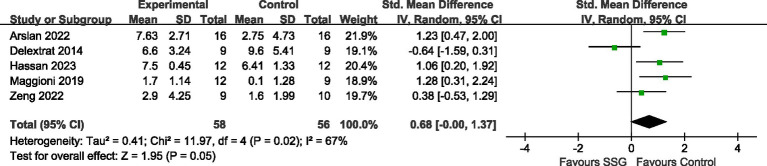
Forest plot of SSG intervention on passing.

Regarding passing performance, the meta-analysis results also indicated a statistically significant difference between the SSG group and the control group (SMD = 0.68, 95% CI: −0.00 to 1.37, *p* = 0.05) (Figure 9).

#### Dribbling

**Figure 10:**
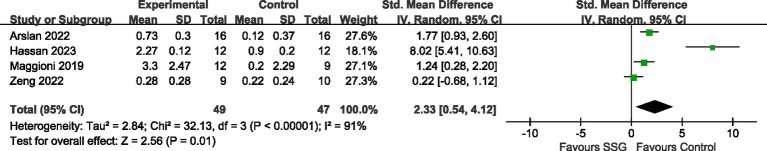
Forest plot of SSG intervention on dribbling.

Regarding dribbling, the meta-analysis results also showed a statistically significant difference between the SSG group and the control group (SMD = 2.23, 95% CI: 0.54 to 4.12, *p* = 0.01) (Figure 10).

### Subgroup analysis according to control intervention type (HIIT vs. TBT)

#### Aerobic performance

**Figure 11:**
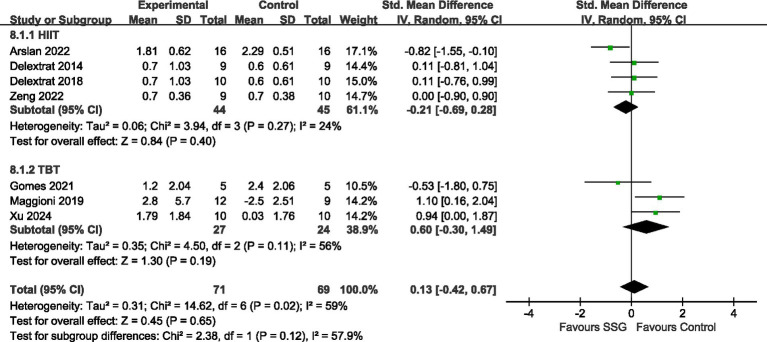
Forest plot of subgroup analysis of SSG intervention on aerobic performance according to control intervention type (HIIT vs. TBT).

Subgroup analysis indicated that SSG did not produce statistically significant improvements in aerobic performance when compared with either HIIT or TBT (Figure 11). Although the effect estimate in the TBT subgroup (SMD = 0.60, 95% CI = −0.30 to 1.49, *p* = 0.19) appeared more favorable than that in the HIIT subgroup (SMD = −0.21, 95% CI = −0.69 to 0.28, *p* = 0.40), neither comparison reached statistical significance.

#### Sprinting

**Figure 12:**
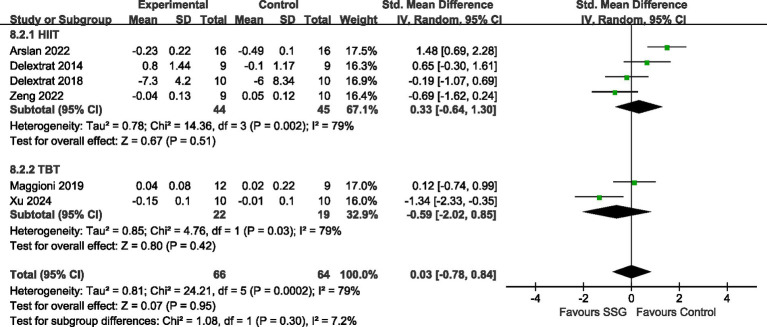
Forest plot of subgroup analysis of SSG intervention on sprinting according to control intervention type (HIIT vs. TBT).

Subgroup analysis showed no significant improvement in sprinting when SSG was compared with either HIIT (SMD = 0.33, 95% CI = −0.64 to 1.30, *p* = 0.51) or TBT (SMD = −0.59, 95% CI = −2.02 to 0.85, *p* = 0.42) (Figure 12), suggesting that the absence of a sprint-related advantage was generally consistent across control intervention types.

#### Jumping

**Figure 13:**
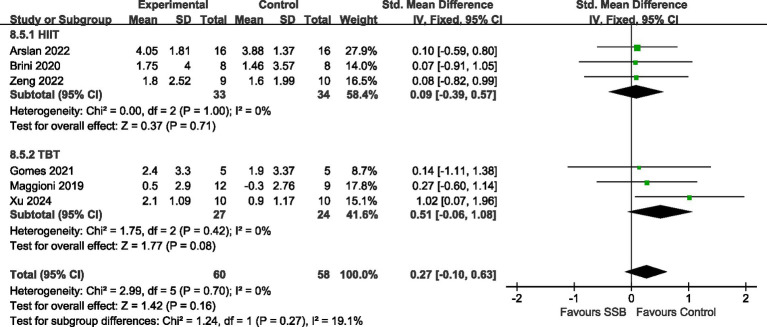
Forest plot of subgroup analysis of SSG intervention on jumping according to control intervention type (HIIT vs. TBT).

Subgroup analysis showed that, compared with the HIIT group (SMD = 0.09, 95% CI = −0.39 to 0.57, *p* = 0.71), SSG did not produce a statistically significant difference in dribbling performance. Similarly, no statistically significant difference was observed when SSG was compared with the TBT group (SMD = 0.51, 95% CI = −0.06 to 1.08, *p* = 0.08) (Figure 13).

#### Agility

**Figure 14:**
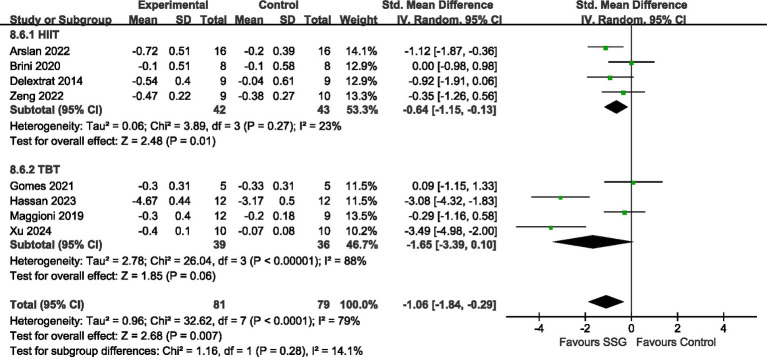
Forest plot of subgroup analysis of SSG intervention on agility according to control intervention type (HIIT vs. TBT).

Subgroup analysis demonstrated that SSG produced a significant improvement in agility when compared with HIIT (SMD = −0.64, 95% CI = −1.15 to −0.13, *p* = 0.01), whereas no statistically significant difference was observed when SSG was compared with TBT (SMD = −1.65, 95% CI = −3.39 to 0.10, *p* = 0.06) (Figure 14).

#### Shooting

**Figure 15:**
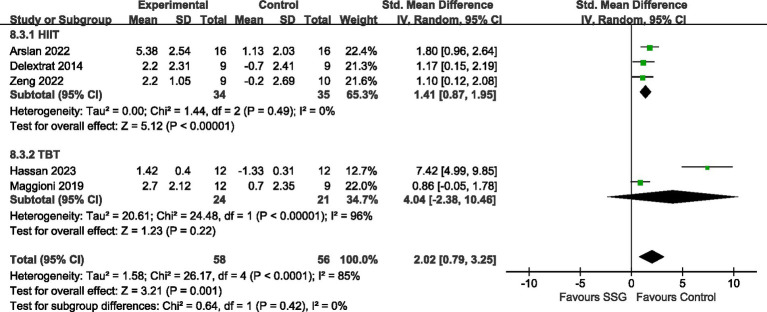
Forest plot of subgroup analysis of SSG intervention on shooting according to control intervention type (HIIT vs. TBT).

The SSG showed a significant advantage over HIIT (SMD = 1.41, 95% CI = 0.87 to 1.85, *p* < 0.01) for shooting, whereas no statistically significant benefit was found relative to TBT (SMD = 4.04, 95% CI = −2.38 to 10.46, *p* = 0.22) (Figure 15). Interpretation of the TBT subgroup should remain cautious because only two studies contributed to that comparison.

#### Passing

**Figure 16:**
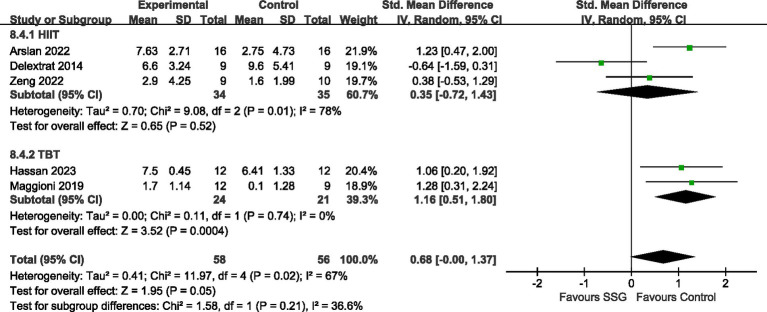
Forest plot of subgroup analysis of SSG intervention on passing according to control intervention type (HIIT vs. TBT).

No statistically significant difference in passing was observed between SSG and HIIT (SMD = 0.35, 95% CI = −0.72 to 1.43, *p* = 0.52); in contrast, a significant improvement was found when SSG was compared with TBT (SMD = 1.16, 95% CI = 0.51 to 1.80, *p* < 0.01) (Figure 16). Nevertheless, this result should be viewed as preliminary, as the TBT subgroup was derived from a limited evidence base.

#### Dribbling

**Figure 17:**
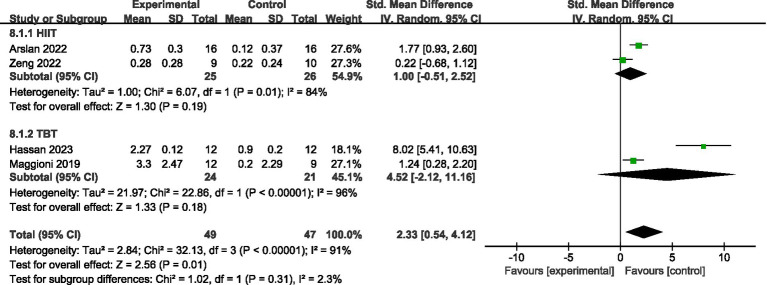
Forest plot of subgroup analysis of SSG intervention on dribbling according to control intervention type (HIIT vs. TBT).

Subgroup analysis showed that, compared with the HIIT group (SMD = 1.00, 95% CI = −0.51 to 2.52, *p* = 0.19), SSG did not produce a statistically significant difference in dribbling performance. Similarly, no statistically significant difference was observed when SSG was compared with the TBT group (SMD = 4.52, 95% CI = −2.12 to 11.16, *p* = 0.18) (Figure 17). This may be related to the limited number of studies included in the analysis.

## Discussion

This systematic review and meta-analysis compared the effects of small-sided games (SSG) and traditional training on athletic performance in basketball players. The results indicated that, compared with the control group, SSG were more effective in improving agility, shooting, passing and dribbling. However, no clear advantages were observed for aerobic performance, sprinting, or jumping. Overall, these findings provide moderate to low certainty evidence regarding the effects of SSG on athletic performance in basketball players.

The findings of the meta-analysis indicated that there were no significant differences in the enhancements of aerobic performance, sprinting, and jumping between the SSG group and the control group. This observation could be partly attributed to the strong physiological stimulus already provided by the training regimen of the control groups (Cao et al., 2024). In the studies analyzed, the control groups primarily engaged in high-intensity interval training or traditional basketball-specific training, which have both demonstrated effectiveness in improving aerobic performance, sprint speed, and explosive power in the lower limbs; this may have lessened the relative benefits of SSG to some extent (Cao et al., 2025; Čaprić et al., 2025). Furthermore, SSG training mainly focuses on simulating game situations and developing technical skills specific to the sport (de Souza et al., 2024). Although the physiological demands of SSG are tailored to the sport, they might not offer sufficiently concentrated stimuli needed to further enhance these physical performance measures.

To better understand whether the effects of SSG differ based on the control conditions, we performed a detailed subgroup analysis focused on the type of control intervention (HIIT vs. TBT). The findings from the subgroup analysis suggest that the relative effects of SSG are influenced, at least in part, by the characteristics of the control intervention; however, this influence is specific to the outcomes measured rather than being uniformly applicable across all cases. Notably, in the comparison between SSG and HIIT, we observed marked improvements in agility and shooting performance, while no significant differences emerged regarding aerobic performance, sprinting, jumping, passing, or dribbling. This approach is logical since HIIT delivers substantial physiological stimulus, potentially diminishing variations in fitness-related outcomes, whereas SSG provide enhanced benefits in movement flexibility and the execution of sport-specific skills under conditions simulating actual matches. The frequent accelerations, decelerations, and changes in direction typical of small-sided training play a crucial role in enhancing players’ agility, while the improvement in shooting performance can likely be linked to the increase in shooting opportunities and specific scoring scenarios generated throughout SSG training. In the comparison of SSG and TBT, no notable statistical differences were found between subgroups regarding aerobic performance, sprinting ability, jumping, agility, shooting, or dribbling. Nevertheless, the relatively larger positive effect sizes observed in agility, shooting, and dribbling suggest that SSG may have favorable trends in these areas. Conversely, passing performance revealed a significant advantage for SSG. This observation implies that, in comparison to TBT, SSG may facilitate basketball players in better utilizing various technical and tactical skills on the court, thus improving performance in dynamic interactive situations. However, caution should be exercised when interpreting the subgroup-specific results for TBT. The estimates for several TBT subgroups were based on a limited number of studies, with some subgroups relying on just two studies for pooled estimates. To summarize, the results of the subgroup analyses indicate that the effectiveness of SSG could be influenced not only by its inherent training features but also by the content and intensity of the comparison intervention. Additional high-quality studies with larger participant numbers and more standardized measures are necessary to confirm these subgroup-specific trends.

From a physiological standpoint, enhancements in aerobic performance are contingent upon systematic adaptations in the cardiovascular system and the metabolic functions of skeletal muscles (Farrell and Turgeon, 2023; Zhou et al., 2024). These adaptations include increases in maximal cardiac output, improved capillary density in skeletal muscles, and a rise in mitochondrial biogenesis (Tomanek, 2022; Scott et al., 2024; Bokov and Popov, 2022). Nonetheless, since SSG primarily focus on the practice and cultivation of sport-specific skills, the overall intensity of exercise often varies significantly, exhibiting frequent shifts between high and low intensities (Kisilewicz et al., 2026). Consequently, the energy supply alternates repeatedly between aerobic and anaerobic metabolism, which may not be adequate to generate a stable and sustained aerobic stimulus throughout the training period, thus restricting advancements in aerobic performance. Improvements in performance related to sprinting and jumping largely depend on specific adaptations within the neuromuscular system. These adaptations are intricately linked to the effective recruitment of motor units, increased neural drive, and enhancements in the elastic characteristics of the muscle-tendon complex (Beausejour et al., 2024; Nikolaidou et al., 2025). Typically, high-intensity, well-structured, and consistently applied explosive stimuli are necessary for achieving these adaptations, which usually require focused lower-limb training of high intensity (Ramírez-delaCruz et al., 2022). On the other hand, activities like acceleration, jumping, and direction changes in small-sided games (SSG) are often influenced more significantly by tactical situations and player interactions, leading to substantial fluctuations in movement intensity. As a result, the high-intensity stimulus provided by SSG is often limited, and these games infrequently include targeted resistance training for the lower limbs, potentially accounting for the observed lack of notable improvements in both sprinting speed and jumping ability (de Souza et al., 2024).

The present meta-analysis observed a superior effect of SSG on agility, which may be attributed to the sport-specific movement demands inherent in small-sided game formats. Compared with traditional training, SSG requires players to frequently perform accelerations, decelerations, rapid changes of direction, and transitions between offensive and defensive actions within limited basketball court space. These repetitive, high-frequency composite movements closely align with the demands of competitive basketball and are considered core determinants of agility performance in basketball players (Wang et al., 2024). SSG provides athletes with a training environment that closely reflects real game situations, ensuring that agility-related actions are consistently integrated with team offensive and defensive tactical intentions, as well as opponents’ behaviors (Zanetti et al., 2022). Under conditions of limited playing space and reduced player numbers, athletes must rapidly adjust their positioning and movement trajectories in response to teammates’ tactical movements, defensive pressure, and changes in spatial availability. This form of “purposeful and strategic movement” emphasizes not only movement speed but also the timing of action transitions and the appropriateness of directional choices (Li et al., 2024). Consequently, the agility developed through SSG more closely corresponds to the “sport-specific agility” required in actual competition, rather than merely pre-planned change-of-direction ability. In addition, SSG substantially increases each player’s ball possession and raises the frequency of offensive-defensive transitions, allowing rapid switches between attacking and defensive roles. Frequent role changes require athletes to integrate technical execution with tactical decision-making, producing rapid responses during penetration, defensive shuffling, or help-defense positioning. This process strengthens agility in complex game situations. Compared with traditional physical conditioning or isolated change-of-direction drills, SSG more effectively develop athletes’ immediate response capabilities in dynamic, unpredictable environments. Finally, the high-frequency changes of direction and short-distance movements elicited by SSG can repeatedly stimulate the stretch-shortening cycle of the lower limb muscles, thereby enhancing the nervous system’s adaptability to multidirectional movement patterns (Yılmaz et al., 2025). The interaction between these neuromuscular adaptations and the tactical decision-making demands may jointly contribute to the significant improvements observed in agility performance.

In terms of basketball-specific technical performance, significant improvements in shooting, passing, and dribbling were observed following small-sided games interventions, highlighting the important value of SSG in basketball technical and tactical training. Small-sided 2v2, 3v3, and 4v4 tactical drills are conducive to refining fundamental skills and developing off-ball movement awareness (Mahyudi et al., 2025). On the one hand, these formats substantially increase individual ball possessions, passing frequency, and shooting opportunities, allowing technical skills to be repeatedly practiced in higher-density and more game-representative contexts. On the other hand, small-number competitive situations require players to make rapid decisions and execute skills within a limited time and space, thereby enhancing the stability and applicability of technical actions in matches (Marín et al., 2025; Tognozzi Jamkosián, n.d.). In addition, the defensive pressure and frequent offensive–defensive transitions commonly present in SSG help players consolidate various offensive and defensive technical details under conditions closely resembling real competition, effectively integrating individual skills into the overall tactical system and ultimately promoting comprehensive improvements in basketball-specific technical performance.

The findings of this review have several practical implications for basketball coaches, strength and conditioning specialists, and sports performance teams. Firstly, SSG appears to be particularly effective in enhancing agility and basketball-specific technical performance, while demonstrating more limited effects on improving aerobic endurance, sprinting, and jumping. Therefore, from a practical application perspective, SSG should primarily be regarded as a training method aimed at enhancing sport-specific skills, assisting players in improving technical and tactical execution under game-simulated conditions, rather than serving as an independent strategy for comprehensive physical development. Based on the characteristics of the included interventions, a feasible implementation approach is to incorporate SSG into structured training programs approximately 2–3 times per week, with session duration varying according to the primary objective of each training session. Player configurations were mainly based on 2v2 and 3v3 formats, which may help increase individual ball contacts, create more technical and tactical decision-making opportunities, and enhance the frequency and repetition of sport-specific actions. When coaches aim to improve players’ agility, shooting, passing, dribbling, and other basketball-specific abilities in a time-efficient manner, this type of training format may be particularly effective. At the same time, the present findings suggest that SSG should not replace traditional physical conditioning when the primary goal is to improve aerobic performance, sprinting, jumping, or broader fitness qualities. Instead, depending on the specific training objective, more favorable outcomes may be achieved when SSG is combined with structured strength and conditioning methods, such as resistance training, plyometric training, or HIIT-based conditioning. For example, coaches may integrate SSG with at least two weekly sessions of neuromuscular or strength-power training to ensure adequate development of explosive capacity and sport-specific physical fitness, while still preserving the training benefits associated with game-based practice. Additionally, it should not be overlooked that the practical application of SSG should also take into account the specific training context, including player age, competitive level, season phase, and the design of the task itself. Adjustments to player numbers, court dimensions, bout duration, recovery intervals, and rule constraints may substantially influence the technical, tactical, and physiological training effects of SSG. Therefore, coaches should individualize the design of SSG according to the intended training outcome rather than uniformly applying a single format across all situations. However, given that the current evidence remains limited and the included studies showed considerable variability in intervention design, these recommendations should be interpreted with caution. Additionally, it is worth noting that the heterogeneity observed across outcomes may be partly attributed to differences in the design features of SSG. First, court dimensions are likely to influence the balance between technical involvement and physiological load, as smaller spaces generally increase players’ movement density, ball contact frequency, and rapid decision-making demands, whereas larger spaces may allow greater running distances. Second, player configuration (e.g., 2v2 or 3v3) may simultaneously affect ball involvement and tactical complexity, thereby altering the relative emphasis on technical execution, agility, and intermittent running demands. Third, training frequency and total intervention duration may influence the extent of players’ physiological and psychological adaptation, as interventions with greater cumulative training loads may provide more opportunities for the acquisition and refinement of sport-specific skills, as well as more frequent neuromuscular stimulation.

This review has several limitations that need to be recognized. To begin with, noticeable heterogeneity was observed in several outcome measures. This heterogeneity may be attributed to differences in participant age, competitive level, SSG formats, court dimensions, intervention duration, and variations in control training protocols across studies; therefore, the findings should be interpreted with caution. Additionally, the relatively small number of included trials and participants (nine RCTs involving approximately 180 players) may have limited the ability of the meta-analysis to detect small to moderate effects for some outcomes. Therefore, non-significant findings should be interpreted with caution, as false-negative conclusions cannot be excluded. Third, the literature reviewed was confined to peer-reviewed publications in English, which might have led to possible publication and language biases. Fourth, the included studies varied considerably in small-sided game formats, court dimensions, work-to-rest ratios, and rule modifications. These differences in task design and contextual constraints may have contributed to between-study heterogeneity and influenced the magnitude of observed effects. Furthermore, because fewer than 10 studies were available for most outcomes, formal statistical tests for publication bias (e.g., Egger’s test) could not be reliably performed. Future studies ought to focus on implementing high-quality randomized controlled trials that utilize standardized training protocols for SSG along with well-defined training loads. Additionally, further exploration is necessary to investigate the relationships between varying SSG factors, such as player count, court dimensions, and work to rest ratios, and specific athletic performance metrics. Moreover, long-term research is essential to determine whether the performance improvements derived from SSG translate into meaningful enhancements in competitive match performance.

## Conclusion

The results of this study suggest that, compared with traditional training, SSG is associated with significant improvements in agility, shooting, passing, and dribbling performance in basketball players, whereas no clear advantages were observed for aerobic performance, sprinting, or jumping. From a practical perspective, SSG may be incorporated into basketball training programs as a sport-specific method to enhance technical and tactical performance, particularly when implemented 2–3 times per week in reduced-player formats such as 2v2 or 3v3. However, when the goal is to optimize broader physical performance, SSG should be integrated with, rather than substituted for, traditional strength and conditioning methods. Overall, SSG may serve as a valuable complementary training strategy, but its prescription should be tailored to the athlete’s specific performance objectives and individual context.

## Data Availability

The original contributions discussed in this research can be found in the article or Supplementary material; any additional questions should be directed to the corresponding author.
