# Supplementary material for: The impact of small-sided games on the athletic performance of basketball players: a systematic review and meta-analysis of randomized controlled trials
Source: Front Psychol. 2026 Jun 26;17:1799413. doi: 10.3389/fpsyg.2026.1799413 (PMC13349824; doi:10.3389/fpsyg.2026.1799413)
Supplement: Supplementary file 1 [file Data_sheet_1.zip › search strategy/search strategy.docx]

**(("Basketball"[Mesh]) AND (((randomized controlled trial[Title/Abstract]) OR (randomized[Title/Abstract])) OR (placebo[Title/Abstract]))) AND (((((((((SSG[Title/Abstract]) OR (small sided game*[Title/Abstract])) OR (small-sided game*[Title/Abstract])) OR (games based training[Title/Abstract])) OR (games based drill[Title/Abstract])) OR (conditioned game*[Title/Abstract])) OR (modified game*[Title/Abstract])) OR (task constraints game*[Title/Abstract])) OR (sided-game*[Title/Abstract]))**
