# Supplementary figures and images for: The impact of small-sided games on the athletic performance of basketball players: a systematic review and meta-analysis of randomized controlled trials
Source: Front Psychol. 2026 Jun 26;17:1799413. doi: 10.3389/fpsyg.2026.1799413 (PMC13349824; doi:10.3389/fpsyg.2026.1799413)

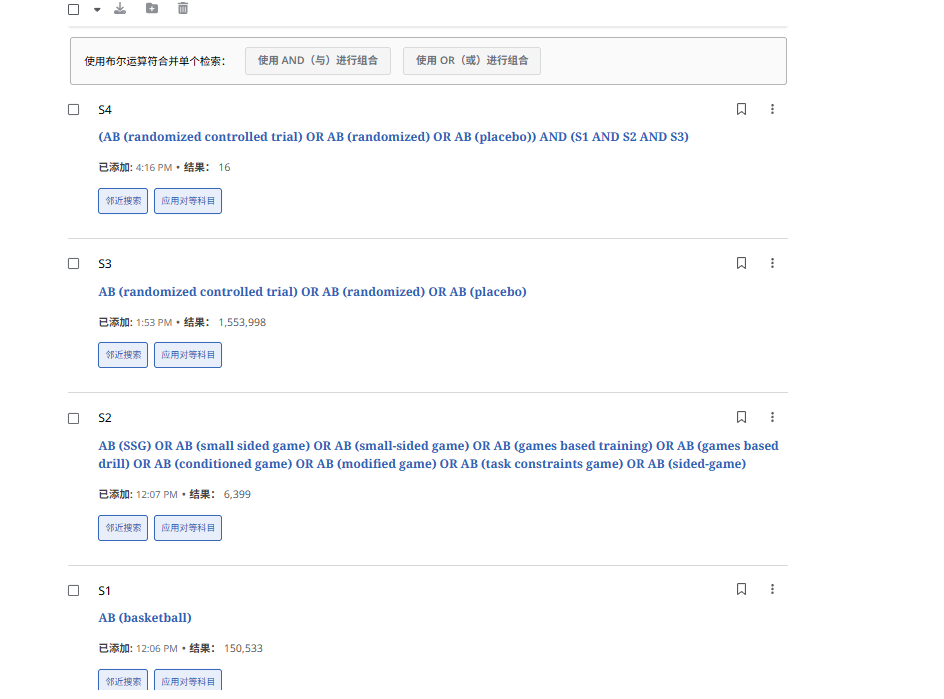

Supplement: Supplementary file 1 [file Data_sheet_1.zip › search strategy/02682b17-b773-4cff-8d20-e9c1586ab90f.png]

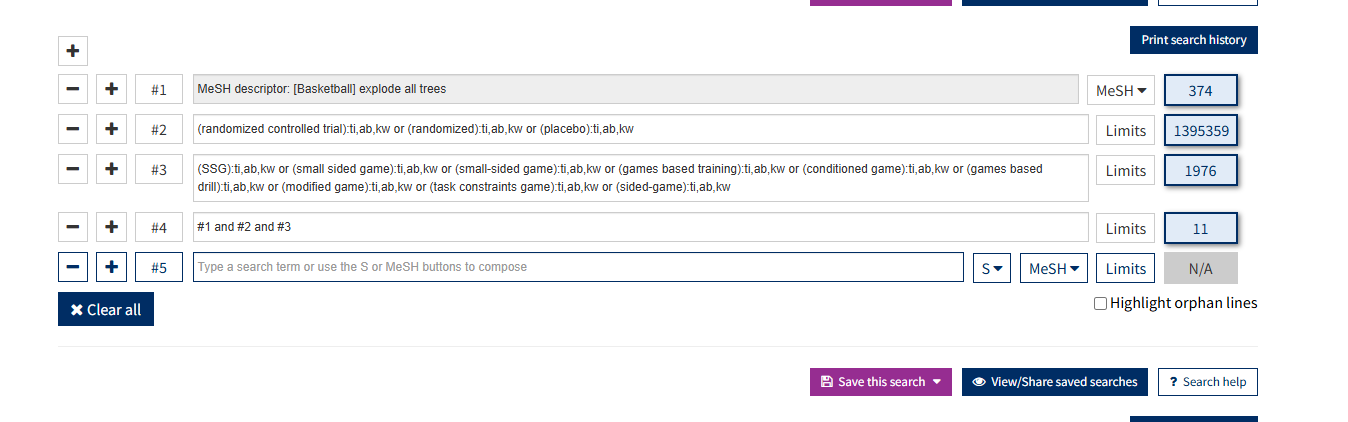

Supplement: Supplementary file 1 [file Data_sheet_1.zip › search strategy/096b106b-82ae-435b-a83b-7ed3481acb17.png]

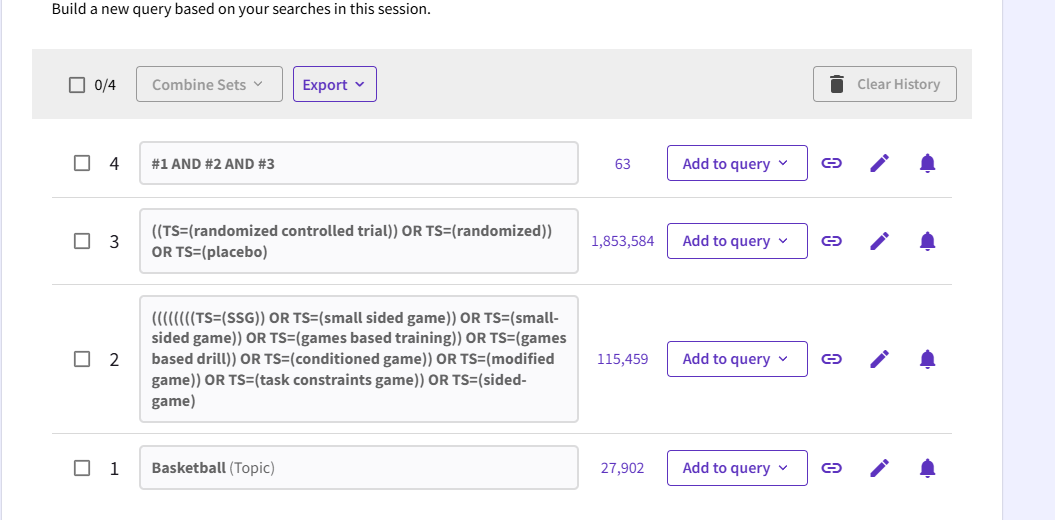

Supplement: Supplementary file 1 [file Data_sheet_1.zip › search strategy/28f14193-28db-4854-bae1-24648312b12c.png]

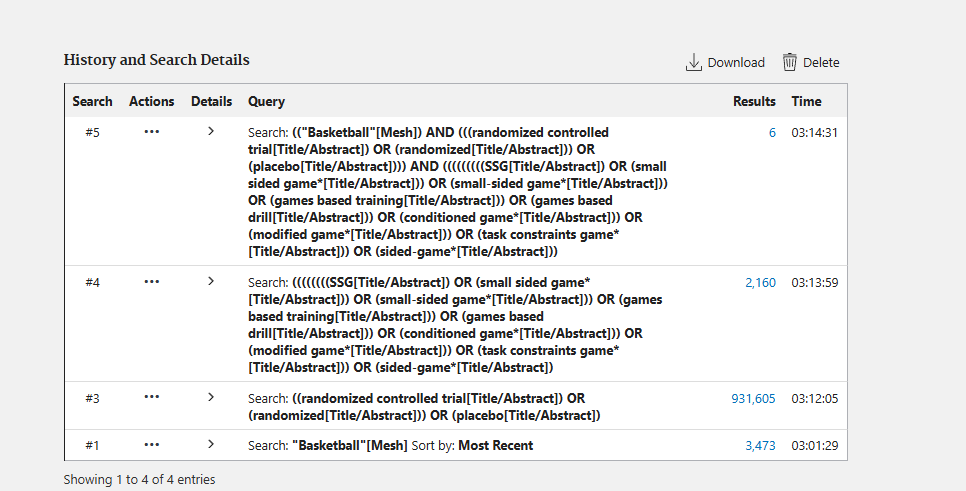

Supplement: Supplementary file 1 [file Data_sheet_1.zip › search strategy/b8b2fff4-3b1a-4547-a8f9-395685e52df3.png]
