# Supplementary material for: The impact of small-sided games on the athletic performance of basketball players: a systematic review and meta-analysis of randomized controlled trials
Source: Front Psychol. 2026 Jun 26;17:1799413. doi: 10.3389/fpsyg.2026.1799413 (PMC13349824; doi:10.3389/fpsyg.2026.1799413)
Supplement: Supplementary file 3 [file Table_2.docx]

### Supplementary Figure 1 – Funnel plot(aerobic)


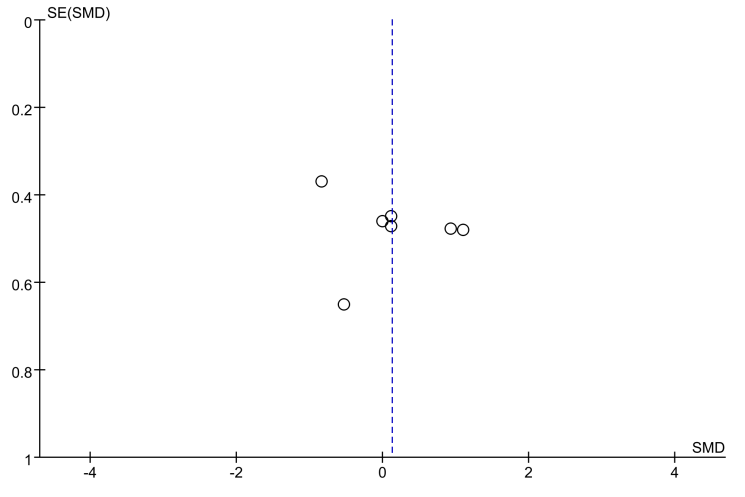


### Supplementary Figure 2 – Funnel plot(agility)


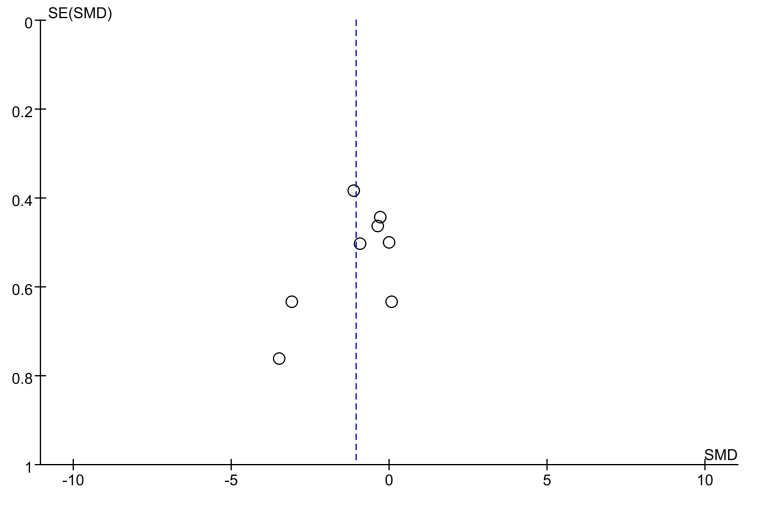


### Supplementary Figure 3 – Funnel plot(dribbling)


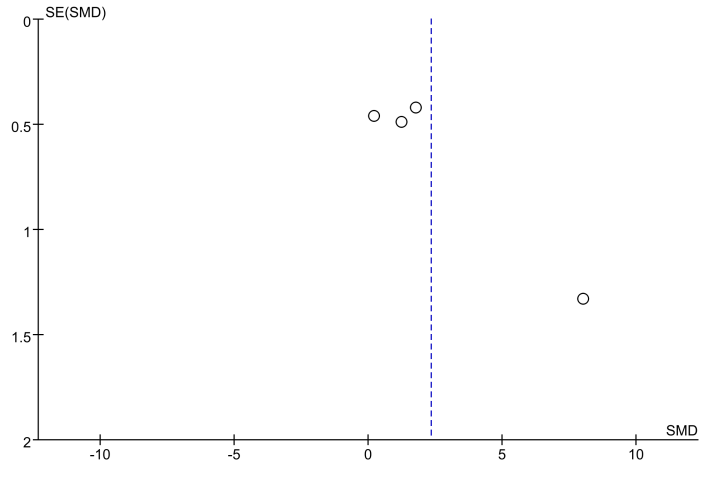


### Supplementary Figure 4 – Funnel plot(jumping)


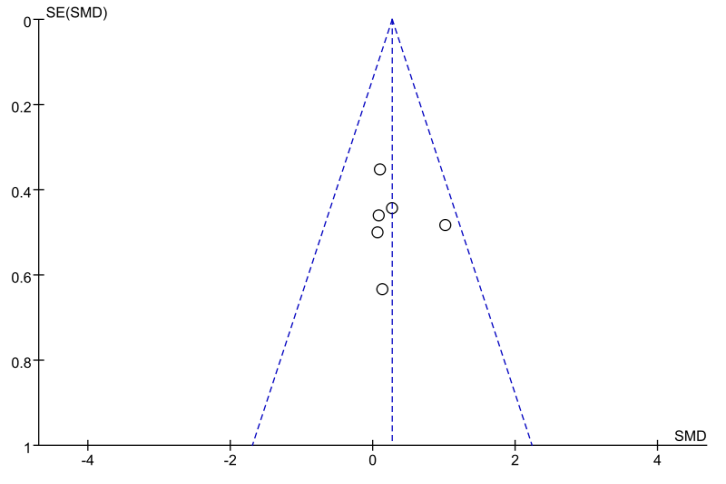


### Supplementary Figure 5 – Funnel plot(passing)


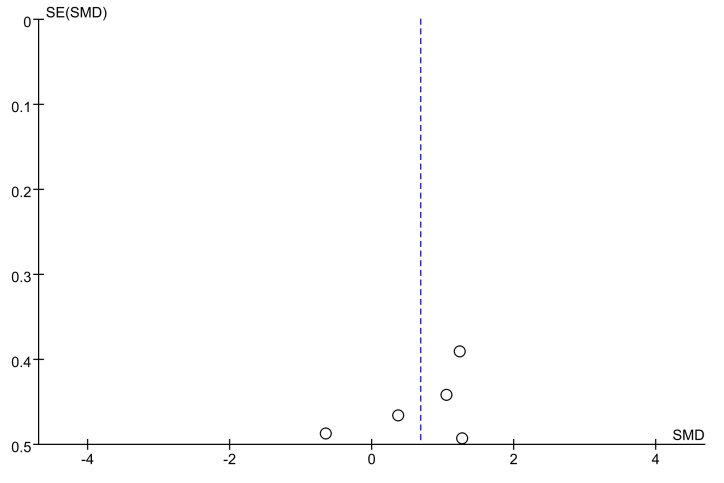


### Supplementary Figure 6 – Funnel plot(shooting)


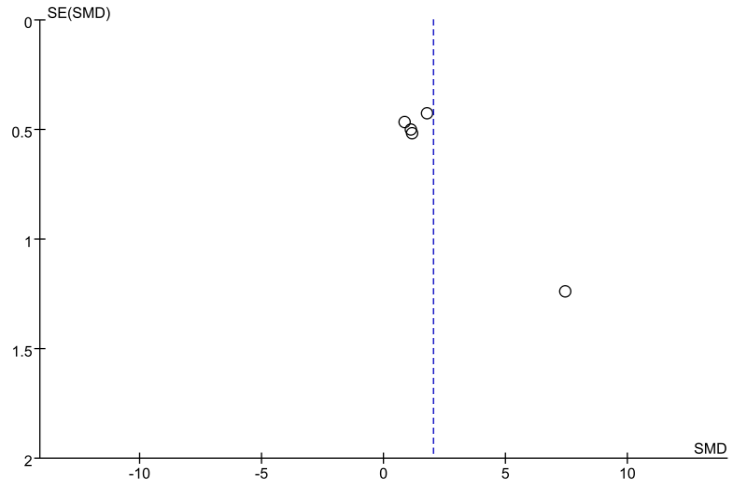


### Supplementary Figure 7 – Funnel plot(sprinting)


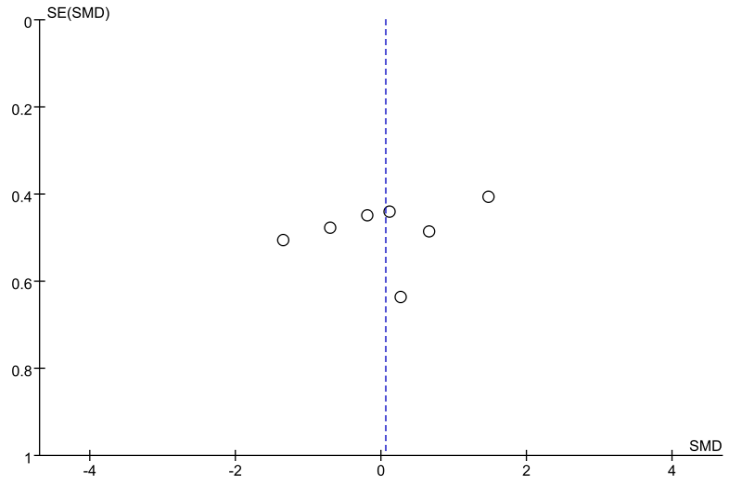


### Supplementary Table 1 – Definition and direction of outcome measures

| **Outcome** | **Measurement Type** | **Unit** | **Better Performance** |
| --- | --- | --- | --- |
| Agility | Time to complete agility test | seconds | Lower is better |
| Sprint | Sprint time (e.g., 20 m) | seconds | Lower is better |
| Jump | Vertical jump height | cm | Higher is better |
| Shooting | Shooting accuracy | % or score | Higher is better |
| Passing | Passing accuracy/score | % or score | Higher is better |
| Dribbling | Time or skill score | seconds / score | Lower or higher depending on test |
