# Supplementary material for: The impact of small-sided games on the athletic performance of basketball players: a systematic review and meta-analysis of randomized controlled trials
Source: Front Psychol. 2026 Jun 26;17:1799413. doi: 10.3389/fpsyg.2026.1799413 (PMC13349824; doi:10.3389/fpsyg.2026.1799413)
Supplement: Supplementary file 4 [file Table_3.docx]

**GRADE Summary of Findings**

*Table S1. GRADE assessment of the certainty of evidence for the effects of small-sided games (SSG) on athletic performance in basketball players*

| **Outcome** | **No. of studies** | **Participants** | **Effect estimate (SMD, 95% CI)** | **Certainty of evidence** | **Reasons for downgrading** |
| --- | --- | --- | --- | --- | --- |
| Aerobic performance | 7 | 140 | 0.13 (-0.42 to 0.67) | ⊕⊕⊕◯ Moderate | Imprecisionᵃ |
| Sprint performance | 7 | 140 | 0.06 (-0.65 to 0.77) | ⊕⊕⊕◯ Moderate | Imprecisionᵃ |
| Agility | 8 | 160 | -1.06 (-1.84 to -0.29) | ⊕⊕⊕◯ Moderate | Inconsistencyᵇ |
| Jump performance | 6 | 118 | 0.27 (-0.10 to 0.63) | ⊕⊕⊕◯ Moderate | Imprecisionᵃ |
| Shooting | 5 | 114 | 2.02 (0.79 to 3.25) | ⊕⊕⊕◯ Moderate | Imprecisionᵃ |
| Passing | 5 | 114 | 0.68 (-0.00 to 1.37) | ⊕⊕◯◯ Low | Imprecisionᵃ, Inconsistencyᵇ |
| Dribbling | 4 | 96 | 2.23 (0.54 to 4.12) | ⊕⊕◯◯ Low | Imprecisionᵃ, Inconsistencyᵇ |

**Footnotes:**

ᵃ Imprecision: downgraded because of the limited number of studies/participants and uncertainty in the pooled estimate.

ᵇ Inconsistency: downgraded because of substantial heterogeneity across included studies.
